# Development of a Novel four-gene Model for Monitoring the Progression from Metabolic Dysfunction-associated Steatotic Liver Disease to Hepatocellular Carcinoma in Males

**DOI:** 10.7150/jca.100724

**Published:** 2025-01-01

**Authors:** Yuchuan Jiang, Jiejian Chen, Lin Xu, Lin Lv, Xiaoning Gan

**Affiliations:** 1 Department of Gastroenterology, The Second Affiliated Hospital, Jiangxi Medical College, Nanchang University, Nanchang, Jiangxi 330000, China.; 2 Department of Medical Oncology, The Second Affiliated Hospital, School of Medicine, South China University of Technology, Guangzhou, Guangdong 510180, China.; 3 Department of Physiology, Michigan State University, East Lansing, MI 48824, USA.

**Keywords:** metabolic dysfunction-associated steatotic liver disease (MASLD), metabolic-associated steatohepatitis (MASH), hepatocellular carcinoma (HCC), differentially expressed genes, diagnostic prediction model

## Abstract

The pathogenesis of metabolic dysfunction-associated steatotic liver disease-associated hepatocellular carcinoma (MASLD-HCC) is complex and exhibits sex-specific differences. Effective methods for monitoring MASLD progression to HCC are lacking. Transcriptomic data from liver tissue samples sourced from multiple public databases were integrated. Utilizing both differential expression analysis and robust rank aggregation analysis, differentially expressed genes (DEGs) in patients with MASLD-HCC were identified. Based on these DEGs, diagnostic prediction models for MASLD (DP.MASLD) and HCC (DP.HCC) were constructed using elastic net analysis for various comparisons, including steatosis versus normal, steatohepatitis versus steatosis, and cancer versus non-cancer. Weighted gene correlation network analysis and gene set enrichment analysis were conducted to unveil the underlying pathogenesis of MASLD-HCC in males. Five overlapping DEGs with diagnostic significance in the progression from MASLD to HCC were identified, namely, *AKR1B10*, *CYR61*, *FABP4*, *GNMT*, and *THBS1*. DP.HCC demonstrated excellent predictive accuracy, with an area under the curve of 0.910 in the training group and 0.981 in the validation group. Similarly, DP.MASLD showed robust predictive accuracy. The pathogenesis of MASLD-HCC in males primarily involves extracellular matrix-receptor interaction, DNA replication, cell cycle, and T-cell receptor signaling. Overall, our study provides a quantitative assessment tool for the early detection and monitoring of MASLD-HCC, highlighting the male-specific molecular characteristics involved in its progression.

## Introduction

Liver cancer is among the most prevalent malignancies globally, with hepatocellular carcinoma (HCC) being the most common histological type of primary liver cancer 1. With the effective prevention and treatment of viral liver diseases, more newly diagnosed patients with HCC are “virus-free” 2. Obesity, alcoholic fatty liver disease, and metabolic dysfunction-associated steatotic liver disease (MASLD) can lead to HCC. Considering the global increase in obesity and type 2 diabetes, MASLD is becoming increasingly prevalent and is an important underlying HCC etiology 3. Recently, differences in the incidence and pathogenesis of MASLD between sexes have received attention. The prevalence and severity of MASLD are higher in males than in premenopausal females 4, 5. A large cohort study of patients with MASLD found that males had a higher MASLD-associated HCC risk than females 6. Globally, each year, the number of new liver cancer cases in males is more than twice that in females 1. Proper consideration of sex differences will provide a better understanding of MASLD-associated HCC pathogenesis and aid in future sex-specific diagnosis and therapy formulation.

Given the high incidence and poor prognosis associated with MASLD-associated HCC, effective methods for early detection remain insufficient. Advances in imaging techniques like computed tomography (CT), magnetic resonance imaging (MRI), and transient elastography (VCTE) have improved the accuracy of liver lesion detection 7, 8. However, these methods are not yet suitable for guiding HCC surveillance 9. Monitoring MASLD-associated HCC and developing corresponding diagnostic and treatment plans remain challenging. Serum biomarkers, such as nucleic acids and proteins, have shown greater potential in tumorigenesis surveillance compared to imaging 10, 11. Although alpha-fetoprotein (AFP) was the first HCC serum biomarker used in clinical practice, its limited specificity and sensitivity underscore the need for novel biomarkers 12, 13. Genomic and proteomic methodologies, combined with machine learning, offer promising opportunities for detecting early indicators and integrating them into routine screening processes, thereby enhancing HCC diagnosis and treatment 12, 14.

In this study, considering the decisive role of sex differences and sample size on model predictive accuracy, transcriptome data from the male cohort in the Gene Expression Omnibus (GEO), ArrayExpress, International Cancer Genome Consortium (ICGC), and The Cancer Genome Atlas (TCGA) databases were screened and integrated to develop approaches for the early diagnosis and surveillance of MASLD-HCC. Furthermore, the main functions and molecular signaling pathways involved in the progression of MASLD to HCC were explored. Our findings will help in the early detection and surveillance of MASLD-HCC progression. Moreover, our findings will reveal the potential underlying pathways that are relevant to the progression from MASLD to HCC.

## Materials and Methods

### MASLD and HCC transcriptome data extraction

The research design of this study is illustrated in a flow diagram (Figure S1). Eligible studies related to MASLD or HCC cohorts were searched and reviewed using the GEO, ArrayExpress, TCGA, and ICGC databases. The search strategy used for MASLD involved: (NAFLD) OR (non alcoholic) OR non-alcoholic) OR nonalcoholic)) AND (fatty liver disease) OR fatty liver) OR fatty livers) OR liver) OR livers) OR steatohepatitis) OR steatohepatitides)) AND “Homo sapiens”. Independent investigators (Xiaoning Gan and Yuchuan Jiang) reviewed and collected the eligible datasets that met the criteria as follows: Inclusion criteria: (i) MASLD diagnosis based on a standardized histopathological assessment system designed by the Pathology Committee of the MASH Clinical Research Network 15; (ii) expression profiling detection in liver tissue samples of male patients; and (iii) availability of original expression profiling data in both steatosis and steatohepatitis specimens. Exclusion criteria: (i) datasets from research on cell lines or animals; (ii) normal liver, steatosis, and steatohepatitis groups with small sample sizes (n < 5); and (iii) expression datasets without transcriptomic data. Moreover, the search strategy and selection criteria to retrieve and extract the eligible datasets of the early-stage HCC male cohort were consistent with those of previous studies 16. Discrepancies between the two investigators were resolved through discussion among all authors. Finally, a total of 372 human liver tissue specimens from male patients with MASLD (GSE48452, GSE61260, GSE89632, and EMEXP3291) and early HCC (GSE76427, GSE84005, TCGA, and ICGC) were included (Table 1).

### MASLD and HCC transcriptome data preprocessing and analysis

Processed data of MASLD and HCC microarray datasets from the ArrayExpress and GEO databases were obtained using R packages ArrayExpress 17 and GEOquery 18, respectively. Microarray probes for each dataset were transformed from probe IDs to Entrez Gene IDs using the R package biomaRt 19. For these microarray probes, if multiple probe IDs were mapped to the same Entrez Gene ID, the Entrez Gene ID expression value was calculated as the median of the probe expression values. RNA-seq datasets of HCC from TCGA and ICGC were extracted using the R package GDCRNATools 20 and the Database of Hepatocellular Carcinoma 21, respectively. The batch effects among these datasets were analyzed using t-distributed Stochastic Neighbor Embedding (t-SNE) analysis and corrected using ComBat in the R package sva 22.

Differential expression analysis (DEA) was performed using the R package limma 23 for MASLD and early-stage HCC datasets. Differentially expressed genes (DEGs) were defined as those with log_2_ fold change (log_2_FC) > 0.5 and *P* < 0.05. The log_2_FC of these DEGs was rescaled to the interval (-5 to 5). Then, the overlapping DEGs from these datasets were analyzed using the robust rank aggregation (RRA) method 24. As the highest-confidence diagnostic predictors for MASLD and HCC, DEGs were further screened with a |log_2_FC| > 2 and an adjusted *P*-value of <0.05 in the RRA analysis. Moreover, intersecting diagnostic predictors in the training datasets of patients with MASLD and HCC were selected using the R package VennDiagram 25.

### DP.MASLD and DP.HCC model construction

The eligible datasets used in the MASLD study were split into training (GSE48452, GSE61260, and EMEXP3291) and validation (GSE89632) groups, similar to those used in the HCC study (training: GSE76427, TCGA, and ICGC; validation: GSE84005). The identified predictors were further analyzed using the elastic net 26 to generate a formula for constructing diagnostic prediction models for MASLD (DP.MASLD) and HCC (DP.HCC). To use the elastic net, the expression data of the identified diagnostic predictors were reduced to genes common to all the merged datasets. The elastic net analysis used the min-cvm penalty to fit a generalized linear model. Leave-one-study-out cross-validation was used for classifier testing in each training dataset, and this classifier was then tested on the validation dataset 27.

### Bioinformatics analyses

Weighted gene correlation network analysis (WGCNA) 28 was utilized to build a weighted gene co-expression correlation network, and the distances between different transcripts were measured using the Pearson correlation coefficient. Construction of the WGCNA network and detection of co-expressed gene modules were conducted using an unsigned topological overlap matrix, β power of 7, and minimum module size of 30. The co-expressed gene modules highly correlated with the characteristics of MASLD-HCC were identified using WGCNA. The gene sets of these modules were analyzed using Gene Set Enrichment Analysis (GSEA) to explore the Gene Ontology (GO) and Kyoto Encyclopedia of Genes and Genomes (KEGG) pathways involved in MASLD progression to HCC 29.

### Hematoxylin-eosin (H&E)

Paired HCC and adjacent non-cancerous liver tissues were collected from ten patients with MASLD-associated HCC at the Second Affiliated Hospital of Nanchang University (Nanchang, China) between January 2023 and June 2024. The study protocol received approval from the ethics committee of the Second Affiliated Hospital of Nanchang University, and informed consent was obtained from all participants. The H&E staining procedure followed the methodology outlined in our previous study 30 and was conducted as follows: Liver tissues from patients with MASLD-associated HCC were fixed in 10% formalin, embedded in paraffin, and sectioned to the appropriate thickness. The sections were deparaffinized in xylene and rehydrated through a graded series of ethanol to water. Staining was performed using Harris hematoxylin for eight minutes and eosin for thirty seconds, followed by dehydration, clearing, and mounting of the slides. All specimens were evaluated and diagnosed by two independent pathologists.

### Quantitative reverse transcription polymerase chain reaction (RT-qPCR)

The RT-qPCR protocol was conducted in accordance with our previous study 30 and proceeded as follows: Total RNA was extracted from liver tissues using TRIzol reagent (Invitrogen, CA, USA) and reverse-transcribed into complementary DNA (cDNA). RT-qPCR was performed using SYBR Green Master Mix (Takara, Kyoto, Japan) on a QuantStudio 5 Real-Time PCR System (Applied Biosystems, USA). The expression levels of target genes were normalized to glyceraldehyde 3-phosphate dehydrogenase (GAPDH). The primer sequences are detailed in Table S1.

### Statistical analyses

Statistical analyses were performed using R (version 4.2.1, http://www.R-project.org), GraphPad Prism (version 8.3.0), SPSS (version 26.0; IBM Corp.), and Microsoft Office 2016 software. The relative expression levels of diagnostic predictors in MASLD and HCC tissues compared with those in normal tissues are represented by the average log_2_FC value. The relative expression levels of these predictors were illustrated using a heatmap. A Student's t-test was used to examine the statistical differences in these diagnostic predictors among the disease groups (steatosis, steatohepatitis, and cancer). Additionally, a paired t-test was used to further validate the differential expression characteristics of these diagnostic predictors between cancerous and adjacent non-cancerous liver tissues in 10 patients with MASLD-associated HCC. Receiver operating characteristic (ROC) curve analysis with the area under the curve (AUC) was performed to assess the diagnostic performances of the DP.MASLD, DP.HCC, and their gene members in male patients with MASLD and HCC using the R package pROC 31.

## Results

### Primary DEGs predicting MASLD progression to HCC

**Figure 1:**
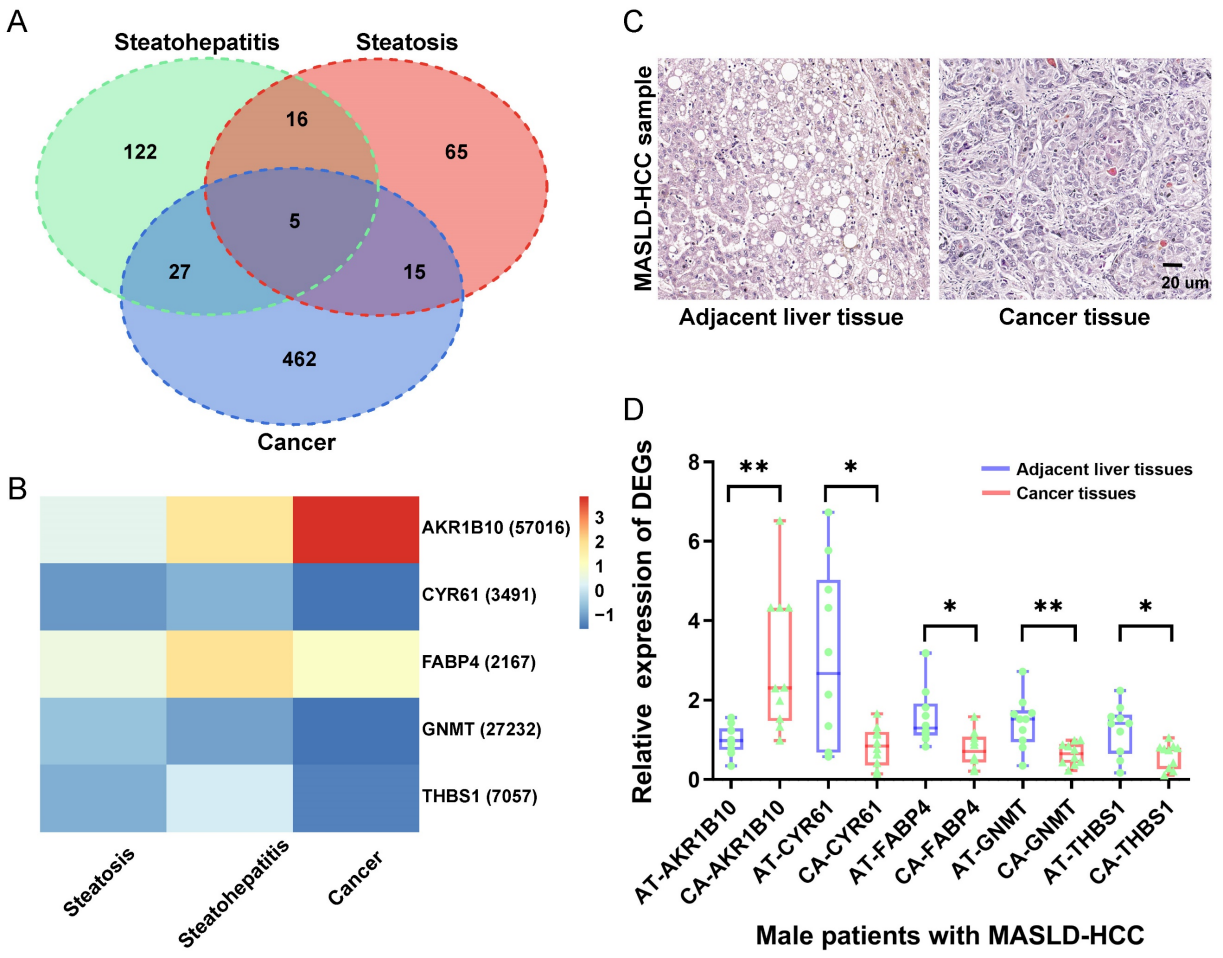
Identification of the expression levels of DEGs in MASLD-associated HCC. (A) As illustrated in the Venn Diagram, five robust DEGs (*AKR1B10*, *CYR61*, *FABP4*, *GNMT*, and *THBS1*) were identified from the intersection of DEGs from the steatosis (steatotic liver tissues versus normal liver tissues), steatohepatitis (steatohepatitic liver tissues versus steatotic liver tissues), and cancer groups (cancerous liver tissues versus non-cancerous liver tissues). (B) Compared with the control group, the relative expression levels of each gene represent the log_2_FC values of the DEGs (*AKR1B10*, *CYR61*, *FABP4*, *GNMT*, and *THBS1*) in the steatosis (steatotic liver tissues versus normal liver tissues), steatohepatitis (steatohepatitic liver tissues versus normal liver tissues) and cancer groups (cancerous liver tissues versus non-cancerous liver tissues). (C) Histopathological section of MASLD-associated HCC and adjacent liver tissue stained with H&E. (D) Compared with the control group (adjacent liver tissues), the relative expression levels of each DEGs (*AKR1B10*, *CYR61*, *FABP4*, *GNMT*, and *THBS1*) in the cancer groups (HCC tissues). DEGs: differentially expressed genes; FC: fold change; HCC: hepatocellular carcinoma; MASLD: metabolic dysfunction-associated steatotic liver disease; RRA: robust rank aggregation. AT: adjacent liver tissue; CA: cancer tissue; HCC: hepatocellular carcinoma; H&E: hematoxylin and eosin; *: *P* < 0.05; **: *P* < 0.01.

By combining the DEA and RRA analyses, 101 DEGs (57 upregulated and 44 downregulated) were identified in steatotic liver tissues compared with normal liver tissues, 170 DEGs (103 upregulated and 67 downregulated) were identified in steatohepatitic liver tissues compared with steatotic liver tissues, and 509 DEGs (172 upregulated and 337 downregulated) were identified in cancerous liver tissues compared with non-cancerous liver tissues (Table S2). Venn diagram analysis showed that the three groups shared five DEGs: *AKR1B10*, *CYR61*, *FABP4*, *GNMT*, and *THBS1* (Figure 1A). And, the relative expression levels of these five DEGs in MASLD-HCC are demonstrated (Figure 1B).

To validate our findings on the molecular features of MASLD- HCC, we analyzed ten cases using both histological and gene expression assays. Histological examination with H&E staining revealed distinct morphological characteristics between steatotic liver tissue and HCC within MASLD samples (Figure 1C). The left panel shows adjacent non-cancerous liver tissue, which exhibits moderate steatosis with hepatocytes containing lipid vacuoles while maintaining a normal cellular structure. In contrast, the right panel illustrates HCC tissue, characterized by increased cell density, nuclear pleomorphism, and prominent nucleoli, all of which indicate malignancy. Furthermore, we conducted RT-qPCR analysis to evaluate the differential expression of the genes *AKR1B10*, *CYR61*, *FABP4*, *GNMT*, and *THBS1* in liver tissues from patients with MASLD-HCC (Figure 1D). The expression patterns of these genes were consistent with large-scale transcriptome data from multiple databases, supporting their involvement in the progression from MASLD to HCC. This comprehensive validation underscores the potential of these genes as diagnostic biomarkers for MASLD-associated HCC.

### DP.MASLD and DP.HCC models predicting MASLD progression to HCC

After adjusting the batch effect among the training datasets (GSE48452, GSE61260, and EMEXP3291) using t-SNE and ComBat (Figure S2), the expression signatures of four DEGs (*AKR1B10*, *FABP4*, *GNMT*, and *THBS1*) were selected to construct the DP.MASLD models (Table 2). *CYR61* was excluded from the elastic net analysis as it was not a DEG in the EMEXP3291 results. The risk score formulae for DP.MASLD are as follows:

DP.MASLD (steatosis vs. normal) = -0.123864 + 3.342406 × expression level of *FABP4* - 1.421809 × expression level of *THBS1* - 1.275545 × expression level of *GNMT* + 0.218167 × expression level of *AKR1B10*;

Risk score formula for DP.MASLD (steatohepatitis vs. steatosis) = -1.038706 + 2.501501 × expression level of *FABP4* - 1.1871 × expression level of *THBS1* + 0.397837 × expression level of *AKR1B10*;

Risk score formula for DP.MASLD (steatohepatitis vs. normal) = -1.162571 + 5.843907 × expression level of *FABP4* - 0.234709 × expression level of *THBS1* - 1.275545 × expression level of *GNMT* + 0.616004 × expression level of *AKR1B10*

For consistency with the gene members in the DP.MASLD model, the DP.HCC model was built based on the expressive signatures of these four predictors: *AKR1B10*, *FABP4*, *GNMT*, and *THBS1* (Table 2). The batch effect among the training datasets (GSE76427, TCGA, and ICGC) was adjusted using t-SNE and ComBat (Figure S3). The risk score formula for DP.HCC was established as follows:

DP.HCC (cancerous versus non-cancerous) = 1.668969 + 0.947173 × expression level of *FABP4* - 1.732848 × expression level of *THBS1* - 0.60475× expression level of *GNMT* + 0.862425 × expression level of *AKR1B10*

**Figure 2:**
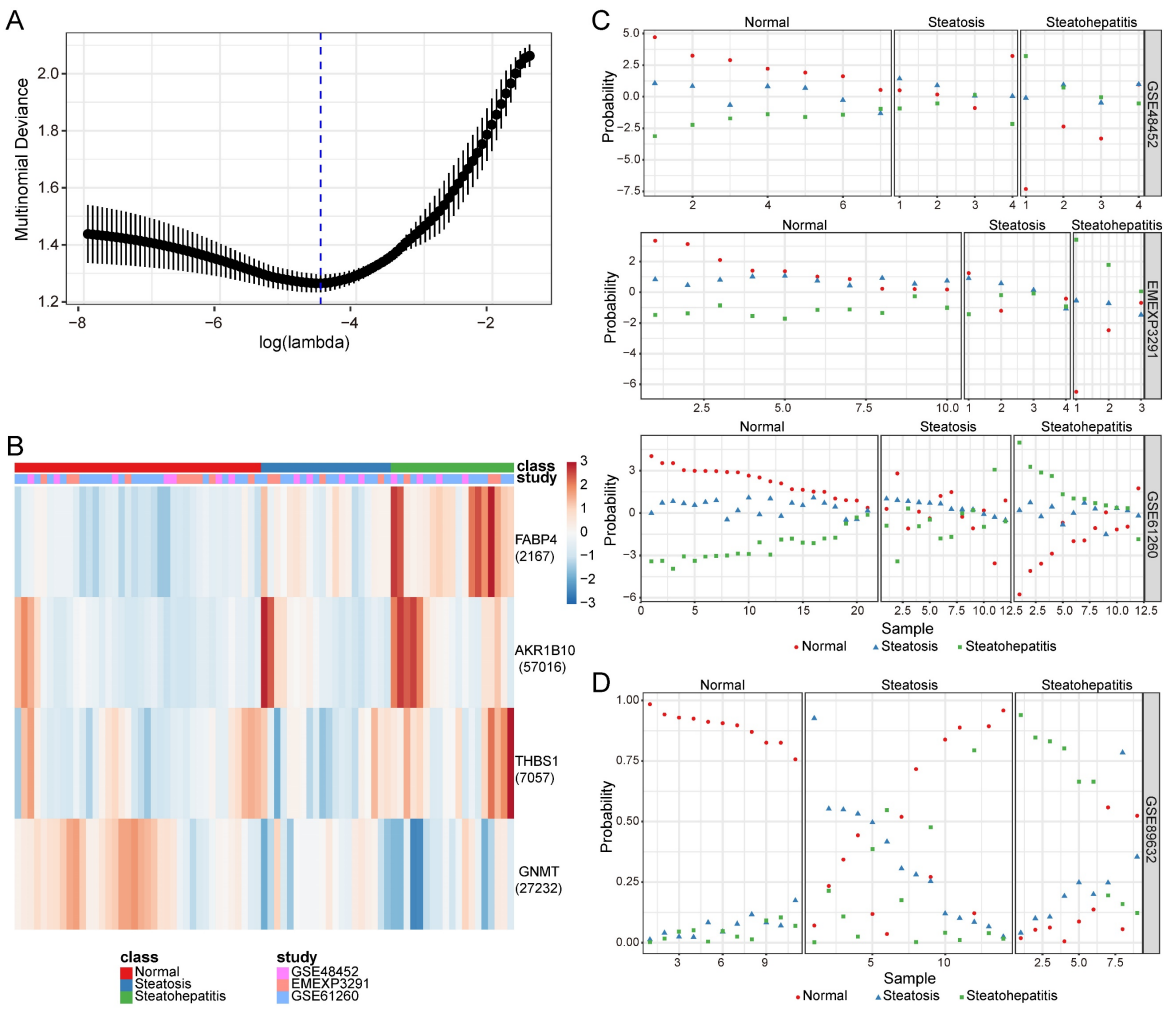
Construction and validation of a four-gene diagnostic classifier for MASLD. (A) Multinomial deviance as a function of the regularization parameter lambda for cross-validation on the training datasets. The dots correspond to the mean, and the error bars correspond to the standard deviation. The coefficients of the four genes were selected using lambda with the minimum multinomial deviance, marked with a blue dashed line (lambda = 0.011, log(lambda) = -4.510). (B) Heatmap describing the expression levels of selected genes in a multinomial classifier erected by the training datasets. Each row represents a gene with its Entrez Gene ID in parentheses, and each column represents a sample. (C) Estimated probabilities for the samples in the training datasets (GSE48452, EMEXP3291, and GSE61260). (D) Estimated probabilities for the samples in the validation dataset (GSE89632). For each sample, three points correspond to the probability that the sample belongs to the respective class. The samples are sorted by true class probability within each dataset and class. For most samples, the probability of the true subtype is close to 1, indicating an unambiguous classification. MASLD: metabolic dysfunction-associated steatotic liver disease.

Based on the lowest multinomial deviance, we identified a multinomial classifier for all the samples from the training datasets (Figure 2A). The heatmap shows the relative expression levels of the four genes (*AKR1B10*, *FABP4*, *GNMT*, and *THBS1*) in normal, steatosis, and steatohepatitis tissues across the three training datasets (Figure 2B). We evaluated the classifier on three independent training datasets (Figure 2C) and one independent validation dataset (Figure 2D) to validate our method. The overall accuracy (fraction of correctly classified samples) of the multinomial classifier for the cross-validation of the training datasets was 79.2%. Across the validation datasets, the overall accuracy was 64.7% (Table S3).

**Figure 3:**
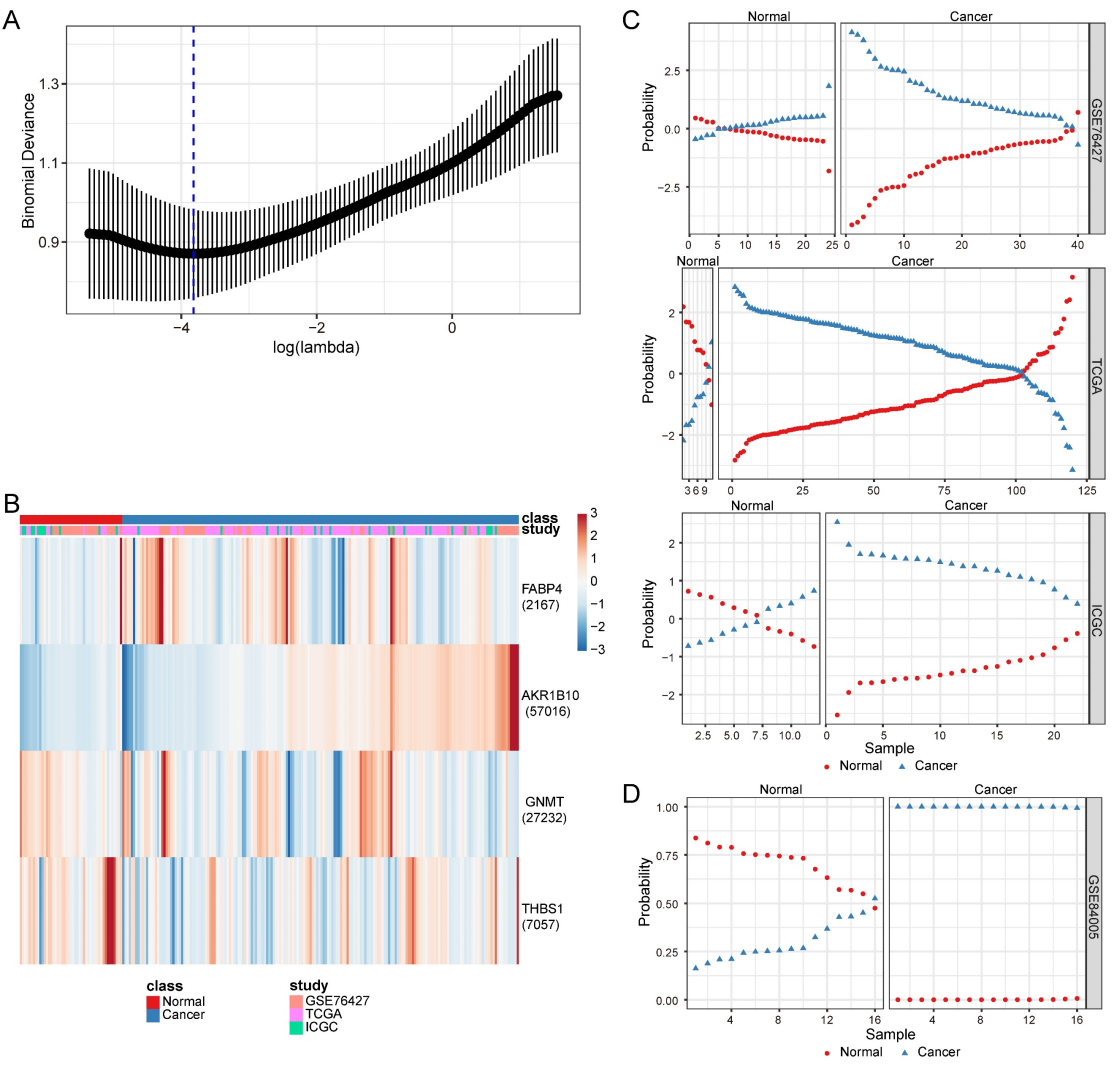
Construction and validation of a four-gene diagnostic classifier for HCC. (A) Binomial deviance as a function of the regularization parameter lambda for cross-validation on the training datasets. The dots correspond to the mean, and the error bars correspond to the standard deviation. The coefficients of the four genes were selected using lambda with minimum binomial deviance, marked with a blue dashed line (lambda = 0.022, log(lambda) = -3.817). (B) Heatmap describing the expression levels of the selected genes in the binomial classifier erected by the training datasets. Each row represents a gene with its Entrez Gene ID in parentheses, and each column represents a sample. (C) Estimated probabilities for samples in the training datasets (GSE76427, TCGA, and ICGC). (D) Estimated probabilities for the samples in the validation dataset (GSE84005). For each sample, two points correspond to the probability that the sample belongs to the respective class. Within each dataset and class, the samples are sorted according to the probability of the true class. For most samples, the probability of the true subtype is close to 1, indicating an unambiguous classification. HCC: hepatocellular carcinoma; ICGC: International Cancer Genome Consortium; TCGA: The Cancer Genome Atlas.

Based on the lowest binomial deviance, we identified a binomial classifier for all samples from the training datasets (Figure 3A). The heatmap shows the relative expression levels of the four genes in cancerous and non-cancerous liver tissues across the training datasets (Figure 3B). To validate our method, we evaluated the classifier using three independent training datasets (Figure 3C) and one independent validation dataset (Figure 3D). The overall accuracy of the binomial classifier for cross-validation of the training datasets was 80.8%. Across the validation datasets, the overall accuracy was 96.9% (Table S4). Thus, we established robust models for predicting MASLD-HCC using transcriptomic data derived from multiple platforms.

### Diagnostic performance of the DP.MASLD and DP.HCC models for predicting MASLD progression to HCC

**Figure 4:**
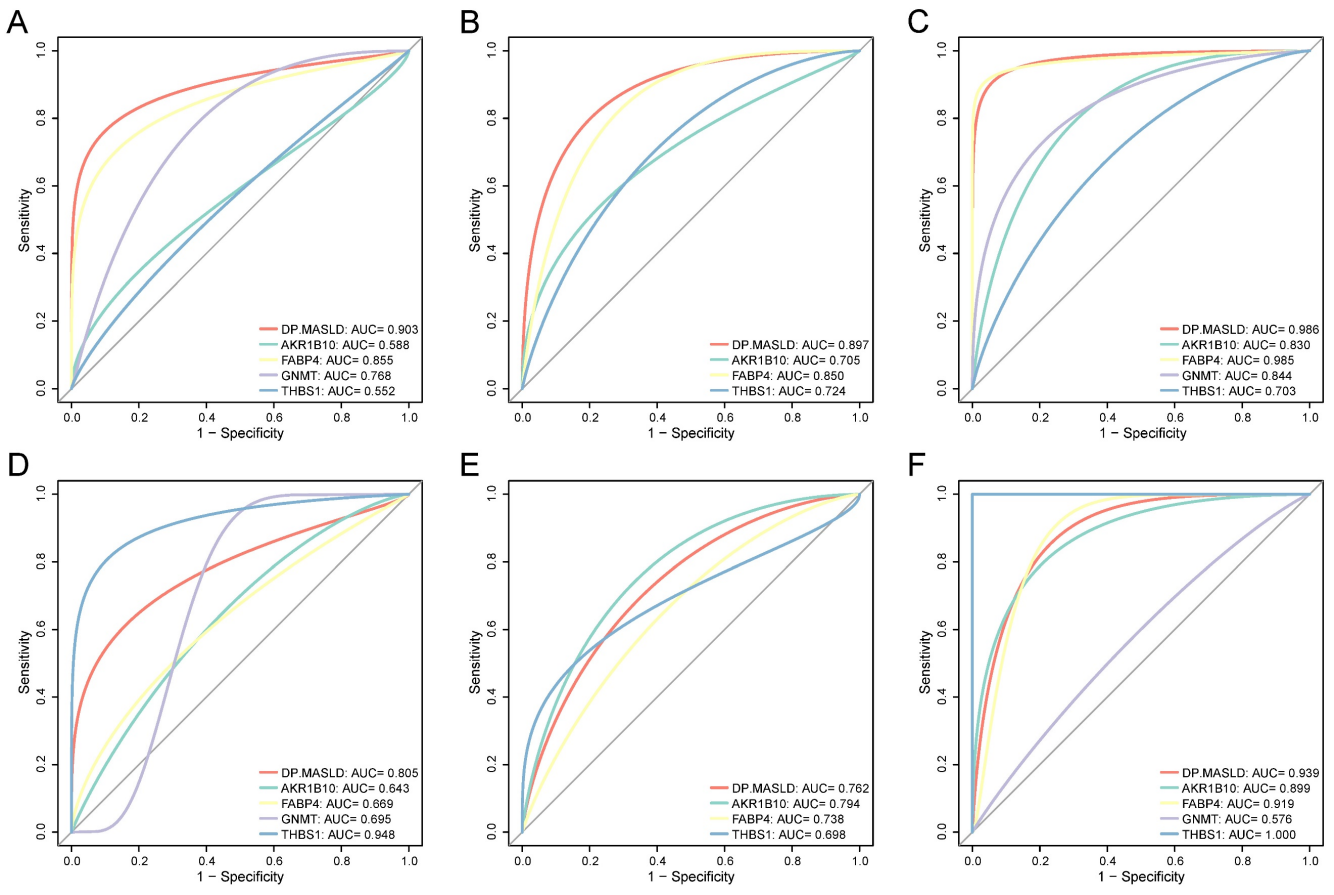
Diagnostic value of the DP.MASLD model and its gene members in MASLD samples. (A) ROC curves of the DP.MASLD model and its gene members for the classification of MASLD (steatosis versus normal) in the training group (GSE48452, GSE61260, and EMEXP3291). (B) ROC curves of the DP.MASLD model and its gene members for the classification of MASLD (steatohepatitis versus steatosis) in the training group. (C) ROC curves of the DP.MASLD model and its gene members for MASLD (steatohepatitis versus normal) in the training group. (D) ROC curves of the DP.MASLD model and its gene members for the classification of MASLD (steatosis versus normal) in the validation group. (E) ROC curves of the DP.MASLD model and its gene members for the classification of MASLD (steatohepatitis versus steatosis) in the validation group. (F) ROC curves of the DP.MASLD model and its gene members for the classification of MASLD (steatohepatitis versus normal) in the validation group. *GNMT* was excluded from the ROC curve analyses used to distinguish steatohepatitis and steatosis because its coefficient was zero in the formula of the DP.MASLD model (steatohepatitis versus steatosis). AUC: area under the curve; DP.MASLD: diagnostic prediction model for MASLD; MASLD: metabolic dysfunction-associated steatotic liver disease; ROC: receiver operating characteristic; 95% CI: 95% confidence interval.

To confirm the diagnostic performance of the DP.MASLD model and its gene members in predicting normal liver, steatosis, and steatohepatitis tissue classification, ROC analyses were performed on MASLD samples in the training group (GSE48452, GSE61260, and EMEXP3291). The AUC of the DP.MASLD model (steatosis versus normal) was 0.903 (95% confidence interval [95% CI]: 0.807-0.998; *P* < 0.001; sensitivity: 70.00%, specificity: 100%, diagnostic threshold value: -5.013; Figure 4A). The AUC of the DP.MASLD model (steatohepatitis versus steatosis) was 0.897 (95% CI: 0.793-1.000; *P* < 0.001; sensitivity: 85.00%, specificity: 89.47%, diagnostic threshold value: 1.443; Figure 4B). The AUC of the DP.MASLD model (steatohepatitis versus normal) was 0.986 (95% CI: 0.793-1.000; *P* < 0.001; sensitivity: 89.47%, specificity: 100.00%, diagnostic threshold value: -3.575; Figure 4C). The results showed that the DP.MASLD model significantly improved the prediction performance over its four-gene signatures alone, including *AKR1B10*, *FABP4*, *GNMT*, and *THBS1* (Figure 4A-C, Table 3).

The predictive performance of the DP.MASLD model and its gene members were verified using the validation group (GSE89632). The DP.MASLD model (steatosis versus normal) AUC was 0.805 (95% CI: 0.626-0.985; *P* = 0.010; sensitivity: 64.28%, specificity: 100.00%, diagnostic threshold value: -6.030; Figure 4D). The DP.MASLD model (steatohepatitis versus steatosis) AUC was 0.762 (95% CI: 0.560-0.964; *P* = 0.038; sensitivity: 57.14%, specificity: 88.89%, diagnostic threshold value: 0.134; Figure 4E). The DP.MASLD model (steatohepatitis versus normal) AUC was 0.939 (95% CI: 0.964-1.000; *P* < 0.001; sensitivity: 77.78%, specificity: 100.00%, diagnostic threshold value: -4.424; Figure 4F). The results showed that although the DP.MASLD model did not achieve the highest accuracy in the validation group; it showed significant improvements and enhanced robustness in diagnosing MASLD compared to its gene members (Figure 4D-F, Table 3).

**Figure 5:**
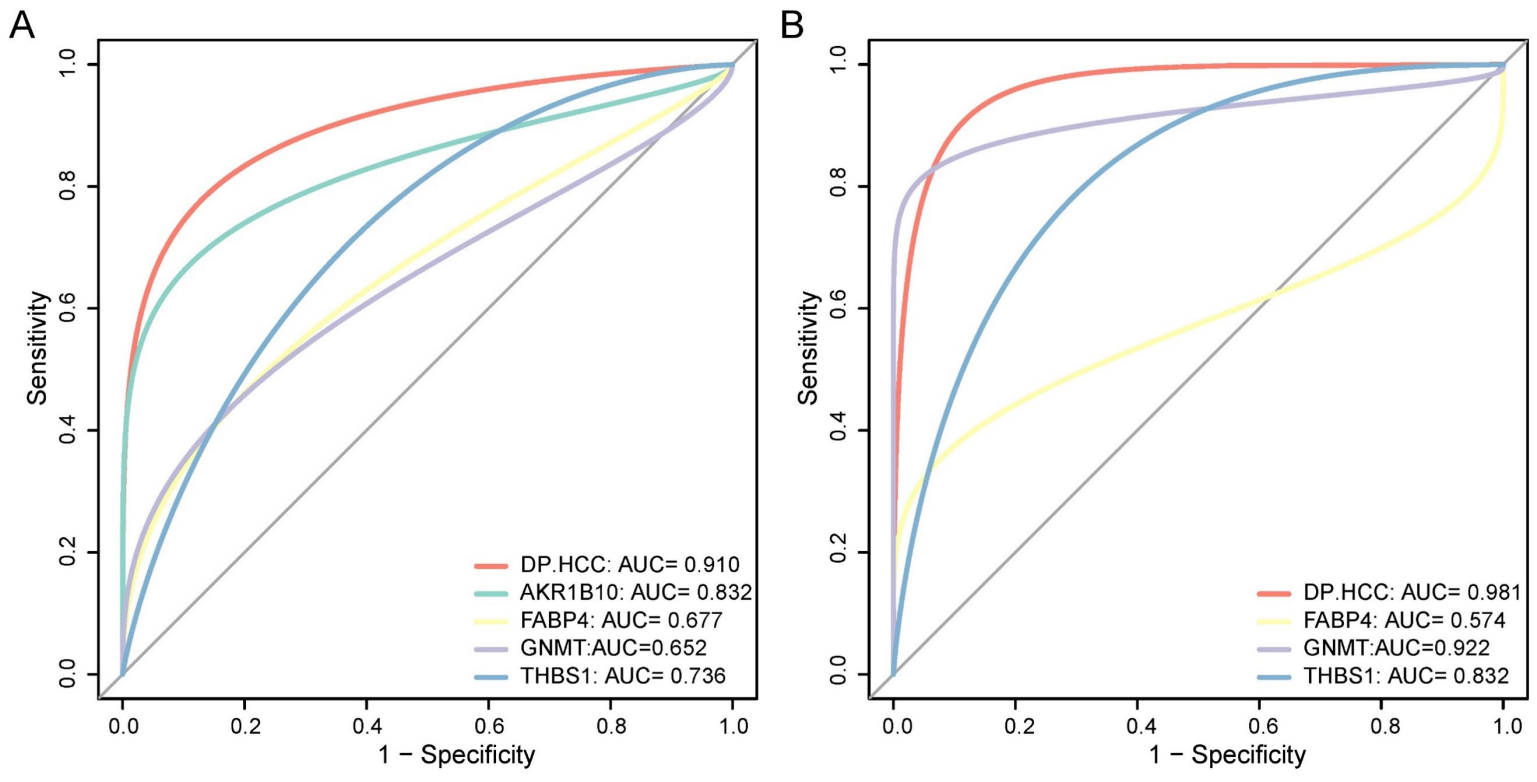
Diagnostic value of the DP.HCC model and its gene members in HCC samples. (A) ROC curves of the DP.HCC model and its gene members for the classification of HCC tissues (cancerous versus non-cancerous) in the training group (GSE76427, TCGA, and ICGC). (B) ROC curves of the DP.HCC model and its gene members for the classification of HCC tissues (cancerous versus non-cancerous) in the validation group (GSE84005). AUC: area under the curve; DP.HCC: diagnostic prediction model for HCC; HCC: hepatocellular carcinoma; ROC: receiver operating characteristic; 95% CI: 95% confidence interval.

In the early-stage HCC training datasets (GSE76427, TCGA, and ICGC), we performed ROC analyses to confirm the predictive performance of the DP.HCC model and its gene members for non-cancerous and cancerous liver tissue classification. The AUC of DP.HCC model for the diagnosis of HCC was 0.910 (95% CI: 0.868-0.952; *P* < 0.001) with a sensitivity of 85.16%, a specificity of 91.49%, and a diagnostic threshold value of -1.236 (Figure 5A). The predictive performance of the DP.HCC model and its gene members was further verified in a validation group of early-stage HCC (GSE84005). The AUC of DP.HCC model for the diagnosis of HCC was 0.981 (95% CI: 0.946-1.000; *P* < 0.001) with a sensitivity of 87.50%, a specificity of 100.00%, and a diagnostic threshold value of -3.622 (Figure 5B). *AKR1B10* was excluded from the ROC curve analysis as its expression data were not available in the GSE84005 dataset matrix file. The results showed that the DP.HCC model significantly enhanced the prediction performance compared to its gene members alone (Table 4). Both the DP.HCC and DP.MASLD models exhibit excellent accuracy and robustness in monitoring MASLD-HCC progression.

### Molecular mechanisms underlying oncogenesis in MASLD progression to HCC

**Figure 6:**
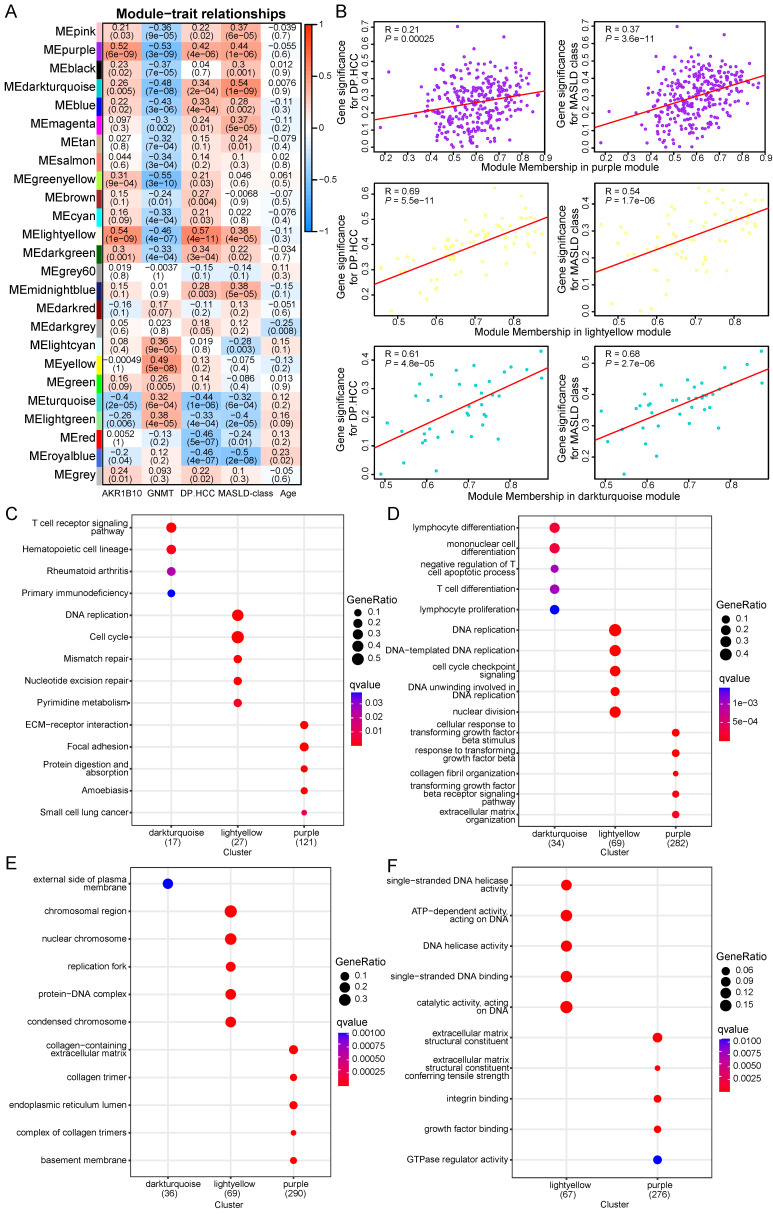
Functional pathways of modules related to oncogenesis from MASLD to HCC. (A) WGCNA showes correlations between module eigengenes and MASLD attributes (*AKR1B10*, *GNMT*, DP.HCC score, MASLD histological class, and age). Each block contains the correlation coefficient and *P*-value. (B) Verification of the correlation between WGCNA gene modules and MASLD traits (DP.HCC score and MASLD histological class). Scatter plots were used to illustrate the correlations of gene significance for traits versus module membership in the dark-turquoise, light-yellow, and purple modules. The Pearson score and *P*-value of each module are shown at the top left of each figure. (C) KEGG gene sets (*P* < 0.05) enriched by co-expressed genes in the WGCNA gene modules of dark-turquoise, light-yellow, and purple. (D-F) Results of GO analysis, including biological process gene sets (*P* < 0.05) (D), cellular component gene sets (*P* < 0.05) (E), and molecular function gene sets (*P* < 0.05) (F), enriched by co-expressed genes in the dark-turquoise, light-yellow, and purple modules. DP.HCC: diagnostic prediction model for HCC; GO: Gene Ontology; HCC: hepatocellular carcinoma; KEGG: Kyoto Encyclopedia of Genes and Genomes; MASLD: metabolic dysfunction-associated steatotic liver disease; WGCNA: Weighted gene correlation network analysis.

Here, we performed WGCNA on a merged expression matrix (GSE48452, GSE61260, GSE89632, and EMEXP3291) of 111 samples from males with MASLD. By setting the soft-thresholding power to seven (scale-free R^2^ = 0.85), we identified 25 modules (Figure S4; non-clustering genes in gray). The correlation coefficients between attributes (*AKR1B10*, *GNMT*, DP.HCC score, MASLD histological class, and age) and eigenvalues of each module are presented in a heatmap (Figure 6A). Gene modules with an absolute total correlation coefficient >1.5 with these attributes (*AKR1B10*, *GNMT*, DP.HCC score, MASLD histological class) were identified from the heatmap. Consequently, we identified purple (300 genes), light-yellow (69 genes), and dark-turquoise (38 genes) modules, all of which were significantly correlated with the DP.HCC score (R = 0.21, *P* < 0.05; R = 0.69, *P* < 0.05; R = 0.61, *P* < 0.05, respectively) and MASLD histological class (R = 0.37, *P* < 0.05; R = 0.54, *P* < 0.05; R = 0.68, *P* < 0.05, respectively) (Figure 6B).

To better understand the molecular mechanisms underlying MASLD progression to HCC, GSEA analysis was performed to analyze the enriched co-expressed genes in the three modules (purple, light-yellow, and dark-turquoise). The co-expressed genes in the three modules were significantly enriched in several KEGG pathways including extracellular matrix-receptor interaction, DNA replication, and T-cell receptor signaling (q-value < 0.05, Figure 6C). GO analysis results indicated that, at the biological process level, the co-expressed genes in these three modules were closely associated with the cellular response to transforming growth factor beta stimulus, DNA replication, and lymphocyte differentiation (q-value < 0.05, Figure 6D).

At the cellular component level, the co-expressed genes were linked to the collagen-containing extracellular matrix, chromosomal region, and external side of plasma membrane (q-value < 0.05, Figure 6E). Regarding molecular function, the co-expressed genes were identified as extracellular matrix structural constituent, single-stranded DNA helicase activity, and ATP-dependent activity acting on DNA (q-value < 0.05, Figure 6F). The pathways enriched by the co-expressed genes in the three modules are closely related to MASLD progression to HCC in male patients (Table S5).

## Discussion

The most common liver disease worldwide is MASLD, characterized by excessive lipid accumulation in hepatocytes. Due to its complex etiology and lack of methods to diagnose MASLD, experts have developed new diagnostic criteria and renamed these conditions metabolic dysfunction-associated fatty liver disease 32. However, the molecular mechanisms underlying pathological fatty liver progression to HCC remain unclear. The increasing incidence of MASLD and the concurrent increase in the number of hepatocellular carcinoma (HCC) cases at a global level is a matter of concern 33. HCC has several risk factors, of which MASLD and its associated metabolic disturbances are of great interest due to their accelerating rise in incidence worldwide. The HCC annual incidence among patients with MASLD is approximately 1.8 per 1,000 person-years 34. MASLD, a metabolic inflammation-based liver disease, shows a sex-specific prevalence with a higher incidence in males than females 35. Compared with females, males exhibit increased visceral fat deposition, lack estrogen signaling, and tend to synthesize fatty acids for fat storage. Males with MASLD also experience more severe hepatic fibrosis and a higher HCC incidence than females 36, 37. There is an increasing awareness regarding the effects of sex on liver disease and cancer outcomes 38, 39. Therefore, it is necessary to explore non-invasive biomarkers to develop a novel strategy for the early detection of male patients with MASLD-associated HCC 40, 41.

Currently, liver biopsy remains the gold standard for diagnosing MASLD and HCC in clinical practice. However, its invasive nature and various limitations make it impractical for the early diagnosis and monitoring of MASLD-HCC 40, 42. Serum biomarkers such as aspartate aminotransferase (AST), alanine aminotransferase (ALT), and alpha-fetoprotein (AFP) provide valuable insights into liver function, inflammation, and disease risk 43. Nevertheless, these biomarkers have limitations, including a lack of specificity and sensitivity, and they do not offer real-time insights. Therefore, there is a pressing need to enhance existing markers and discover novel markers 43. Molecular diagnostics have introduced personalized medicine, helping doctors understand the relationship between genetics and liver function. This approach facilitates customized diagnosis, disease prediction, and treatment for patients with liver disease 44. Omics technologies, including genomics, transcriptomics, proteomics, and metabolomics, are leading the way in finding new biomarkers, enabling precise diagnosis and personalized care 14. The integration of omics data with advanced algorithms, including machine learning, holds significant promise for identifying novel molecular markers and enhancing personalized monitoring, prevention, and treatment strategies. Recent studies have increasingly employed machine learning to identify accurate biomarkers and molecular diagnostics for MASLD-HCC. Research has demonstrated that the GALAD score model, developed from serum markers and clinical features, is more accurate and reliable for monitoring HCC in patients with chronic liver disease compared to traditional single markers 45. Although this study did not differentiate between the various causes of chronic liver disease, subsequent research indicates that it can also assess HCC risk in patients with MASH 46. Recently, Luis A. Rodriguez *et al.* developed a highly accurate predictive model utilizing electronic health data from over 1.8 million patients with MASLD to differentiate between those with and without HCC. This model serves as a promising starting point for monitoring MASLD-HCC 47, although it does not elucidate the molecular mechanisms underlying MASLD-HCC. Therefore, the novel biomarkers and molecular model we identified based on transcriptomic data are essential for monitoring MASLD-HCC. These findings not only demonstrate high accuracy and reliability across various datasets but also provide a robust foundation for developing tailored clinical strategies for diagnosing, predicting, and treating MASLD-HCC.

In the present study, transcriptomic data of male liver samples from GEO, ArrayExpress, TCGA, and ICGC databases were used to establish prediction models for the early detection and surveillance of MASLD-associated HCC. Five DEGs with diagnostic values for male MASLD-associated HCC were screened: *AKR1B10*, *CYR61*, *FABP4*, *GNMT*, and *THBS1*. The DP.HCC and DP.MASLD models were established using the elastic net method to analyze these DEGs. The AUC of the DP.HCC model in the training and validation datasets was 0.910 and 0.981, respectively. Thus, we established molecular models with robust and reliable accuracy for the quantitative assessment of the MASLD-HCC progression risk in males. The DP.MASLD and DP.HCC models not only demonstrate strong predictive performance but also provide a cost-effective alternative to traditional diagnostic methods. By utilizing non-invasive biomarkers and transcriptomic data, these models can reduce healthcare costs and enhance patient comfort, particularly in high-risk male populations. The increasing availability and affordability of molecular testing platforms, such as RT-qPCR and RNA-seq, further improve the feasibility of implementing these models in clinical practice. They can be seamlessly integrated into routine screening for the early detection of MASLD-associated HCC with minimal disruption to current workflows. Moreover, by integrating these models with electronic health record systems, automated risk assessments could become a reality, leading to timely interventions and improved patient outcomes. Ultimately, we aim to advance the development of novel diagnostic approaches and sex-specific therapies for male patients with MASLD-associated HCC.

The four DEGs (*AKR1B10*, *FABP4*, *GNMT*, and *THBS1*) selected to build the diagnostic models had been previously examined as molecular markers of MASLD and HCC. AKR1B10, a human nicotinamide adenine dinucleotide phosphate (NADPH)-dependent reductase, was upregulated in HCC and MASLD 48. AKR1B10 is a potent marker for differentiating early HCCs from benign hepatic lesions, with better diagnostic performance than AFP 49. Although MASH is diagnosed via biopsy, non-invasive methods are preferable. Serum or plasma AKR1B10 could be a non-invasive biomarker for predicting MASLD progression and HCC development 50. Moreover, the study suggests that the E2F1/AUF1/AKR1B10 axis may represent a potential therapeutic target for HCC 51. Fatty acid-binding protein 4 (FABP4) plays a crucial role in fatty acid transport and is significantly elevated in the serum of patients with MASLD and HCC 52. FABP4 may contribute to carcinogenesis, particularly in the context of underlying obesity 52, 53. Targeting the FABP4-related fatty acid metabolic axis could potentially prevent the progression of MASH to HCC 54. Glycine N-methyltransferase (GNMT) is the most abundant methyltransferase and an important enzyme involved in S-adenosylmethionine catabolism in the liver 55. MASLD development and progression to HCC are characterized by the downregulation of *GNMT*. Loss of liver *GNMT* promotes liver steatosis and the transition to HCC 56. Furthermore, *GNMT* deficiency may impair the efficacy of transarterial chemoembolization (TACE) in HCC treatment by affecting hypoxia signaling and glycolysis pathways 57. Thrombospondin 1 (THBS1/TSP1), a matricellular glycoprotein, modulates various cellular functions by interacting with extracellular proteins and cell-surface receptors. Although its role in liver diseases is not fully understood 58, research indicates that *THBS1* promoter methylation may inhibit tumor angiogenesis in HCC, suggesting *THBS1* as a potential therapeutic target 59. While the diagnostic and therapeutic potential of these biomarkers in MASLD and HCC requires further validation, our findings offer valuable insights for exploring candidate biomarkers for the diagnosis and treatment of MASLD-associated HCC.

It is well-established that MASLD can progress from simple steatosis to steatohepatitis and further develop into HCC 60. However, the specific molecular events in the liver that drive this progression remain poorly understood. Recently, several studies have attempted to explore hub genes and mechanisms involved in the pathological process of MASLD or MASH. For example, using bioinformatic analyses, two research groups identified one consistent hub gene (*AKR1B10*) for the liver steatosis progression to MASH 61, 62. Wu *et al.* conducted WGCNA on two GEO datasets (GSE48452 and GSE89632) and screened 10 potential hub genes in MASLD 63. Immune infiltration analysis has also revealed the pathways related to MASH inflammation 62, 64. However, the hub genes identified in these studies did not distinguish between male and female patients. Additionally, they only analyzed DEGs between MASLD (steatosis or steatohepatitis) and normal liver tissues but not between HCC and non-cancerous tissue samples. In fact, sex, sex hormones, and gender habits affect the risk profiles and phenotypes of liver disease 38, 65. Thus, appropriately considering these aspects could lead to a better understanding of the sex differences in MASLD-HCC risk, molecular characteristics, and therapeutic targets and aid in achieving sex-specific therapies.

In this study, we focused on male patients who are at an elevated risk of developing MASLD-HCC. We investigated the disease progression from normal liver tissue through MASLD to HCC. Utilizing WGCNA, GO functional, and KEGG pathway enrichment analyses, our results demonstrate that the pathogenesis of MASLD-HCC in males is primarily associated with dysregulation in the cell cycle, DNA replication, extracellular matrix-receptor interaction, and T-cell receptor signaling pathways. Notably, the cell cycle checkpoint functions as a critical regulatory mechanism for DNA replication, ensuring the prevention of genetic errors during cell division 66. This underscores the close connection between the cell cycle and DNA replication pathways, where their dysregulation plays a crucial role in occurrence and development of MASLD-HCC 67. Chronic liver inflammation in MASH has been identified as a potential trigger for HCC, even in the absence of cirrhosis 68. T-cell receptor pathways significantly contribute to carcinogenesis and the regulation of liver tumorigenesis in MASLD. For instance, in MASH mouse models, the activation of CD8+ T cells and natural killer T cells has been shown to accelerate tumor development 69. Conversely, CD4+ T cells are essential for effective immune surveillance, reducing the risk of malignant transformation in hepatocytes 70. The selective loss of CD4+ T lymphocytes in MASLD may facilitate HCC progression 71. The tumor microenvironment in HCC is characterized by interactions between various immune cells and non-immune stromal cells, such as cancer-associated fibroblasts and endothelial cells. The extracellular matrix-receptor interaction pathway plays a vital role in shaping the HCC tumor microenvironment 72. Additionally, stromal cells within the tumor microenvironment are key regulators of tumor growth, invasion, and metastasis 73, 74.

Our study had the following limitations: First, the overlapping DEGs between MASLD and HCC screened here may not fully represent the DEGs in MASLD progression to HCC because the HCC samples might include patients with other HCC-associated diseases such as viral hepatitis and alcoholic liver disease. Second, it is crucial to integrate clinical information to enhance the robustness of our model. Future studies that involve the integration of clinical data to further refine and validate our model's predictive capabilities are significant. Third, we focused only on male patients and screened for common DEGs between MASLD and HCC. In the future, the exact roles of DEGs in female patients with MASLD and HCC should be studied accordingly, and the molecular characteristics of MASLD-associated HCC in males and females should be compared.

## Conclusions

Our study established a robust and accurate molecular model for monitoring the progression of MASLD-HCC in male patients, providing a valuable risk assessment tool for disease progression. This tool is expected to enhance early diagnosis, pathological grading and support the molecular classification of MASLD-HCC in clinical practice. Moreover, our research elucidates the key biological functions and molecular pathways involved in the progression from MASLD to HCC, offering critical insights into the underlying molecular mechanisms. These findings may provide valuable insights into the molecular characteristics of MASLD-HCC in males, facilitating the development of sex-specific therapies. Consequently, our study lays a solid theoretical foundation for the future development of molecular diagnostics and targeted therapies specifically designed for male MASLD-HCC patients.

## Supplementary Material

Supplementary figures and tables.

## Figures and Tables

**Table 1:** Summary information of the eligible datasets used in our study.

Dataset	Platform	Disease	Sample size	Sample type (liver tissue)	Sample source
GSE48452	GPL11532	MASLD	Total=73; Male=15; Female=58	Normal=41; Steatosis=14; Steatohepatitis=18	Germany
EMEXP3291	A-AFFY-183	MASLD	Total=45; Male=17; Female=26	Normal=19; Steatosis=10; Steatohepatitis=16	USA
GSE61260	GPL11532	MASLD	Total=109; Male=45; Female=64	Normal=62; Steatosis=23; Steatohepatitis=24	USA
GSE89632	GPL14951	MASLD	Total=63; Male=34; Female=29	Normal=24; Steatosis=20; Steatohepatitis=19	Canada
GSE84005	GPL5175	HCC	Total=36; Male=32; Female=4	Normal=18; Cancer=18	China
GSE76427	GPL10558	HCC	Total=83; Male=64; Female=19	Normal=28; Cancer=55	Singapore
TCGA	TCGA	HCC	Total=187; Male=131; Female=56	Normal=18; Cancer=169	USA
ICGC	HCCDB	HCC	Total=55; Male=34; Female=21	Normal=22; Cancer=33	Japan

HCC: hepatocellular carcinoma; HCCDB: Database of Hepatocellular Carcinoma; ICGC: International Cancer Genome Consortium; MASLD: metabolic dysfunction-associated steatotic liver disease; TCGA: The Cancer Genome Atlas.

**Table 2:** Coefficient of gene signatures in diagnostic models for predicting the MASLD progression to HCC in males.

Gene Symbol (Entrez ID)	Steatosis vs. Normal	Steatohepatitis vs. Steatosis	Steatohepatitis vs. Normal	Cancerous vs. Non-cancerous
Intercept	-0.123864	-1.038706	-1.162571	1.668969
*FABP4* (2167)	3.342406	2.501501	5.843907	0.947173
*THBS1* (7057)	-1.421809	1.1871	-0.234709	-1.732848
*GNMT* (27232)	-1.275545	0	-1.275545	-0.60475
*AKR1B10* (57016)	0.218167	0.397837	0.616004	0.862425

HCC: hepatocellular carcinoma; MASLD: metabolic dysfunction-associated steatotic liver disease.

**Table 3:** Predictive performance of the DP.MASLD model for MASLD tissue classification.

		Steatosis versus Normal					
Variables	Training group				Validation group		
	AUC	95% CI	*P*-value		AUC	95% CI	*P*-value
DP.MASLD	0.903	0.807-0.998	*P* < 0.001*		0.805	0.626-0.985	*P* = 0.010*
*AKR1B10*	0.588	0.418-0.759	*P* = 0.273		0.643	0.416-0.870	*P* = 0.228
*FABP4*	0.855	0.740-0.971	*P* < 0.001*		0.669	0.449-0.889	*P* = 0.155
*GNMT*	0.768	0.646-0.891	*P* < 0.001*		0.695	0.436-0.953	*P* = 0.101
*THBS1*	0.552	0.388-0.716	*P* = 0.518		0.948	0.867-1.000	*P* < 0.001*
		Steatohepatitis versus Steatosis					
Variables	Training group				Validation group		
	AUC	95% CI	*P*-value		AUC	95% CI	*P*-value
DP.MASLD	0.897	0.793-1.000	*P* < 0.001*		0.762	0.560-0.964	*P* = 0.038*
*AKR1B10*	0.705	0.539-0.872	*P* = 0.028*		0.794	0.593-0.994	*P* = 0.020*
*FABP4*	0.850	0.728-0.976	*P* < 0.001*		0.738	0.525-0.951	*P* = 0.059
*GNMT*	N/A	N/A	N/A		N/A	N/A	N/A
*THBS1*	0.724	0.564-0.884	*P* = 0.017*		0.698	0.477-0.920	*P* = 0.115
		Steatohepatitis versus Normal					
Variables	Training group				Validation group		
	AUC	95% CI	*P*-value		AUC	95% CI	*P*-value
DP.MASLD	0.986	0.964-1.000	*P* < 0.001*		0.939	0.840-1.000	*P <* 0.001*
*AKR1B10*	0.830	0.717-0.943	*P* < 0.001*		0.899	0.764-1.000	*P* = 0.003*
*FABP4*	0.985	0.956-1.000	*P* < 0.001*		0.919	0.803-1.000	*P* = 0.002*
*GNMT*	0.844	0.735-0.952	*P* < 0.001*		0.576	0.309-0.843	*P* = 0.569
*THBS1*	0.703	0.559-0.847	*P* = 0.013*		1.000	1.000-1.000	*P* < 0.001*

AUC: area under the curve; 95% CI: 95% confidence interval; MASLD: metabolic dysfunction-associated steatotic liver disease; DP.MASLD: diagnostic prediction model for MASLD. *Statistically significant (*P* < 0.05).

**Table 4:** Predictive performance of the DP.HCC model for HCC tissue classification.

Variables	Training group				Validation group		
	AUC	95% CI	*P*-value		AUC	95% CI	*P*-value
DP.HCC	0.910	0.868-0.952	*P* < 0.001*		0.981	0.946-1.000	*P* < 0.001*
*AKR1B10*	0.832	0.779-0.884	*P* < 0.001*		N/A	N/A	N/A
*FABP4*	0.677	0.604-0.751	*P* < 0.001*		0.574	0.352-0.796	*P* = 0.474
*GNMT*	0.652	0.577-0.727	*P* = 0.001*		0.922	0.808-1.000	*P* < 0.001*
*THBS1*	0.736	0.657-0.815	*P* < 0.001*		0.832	0.687-0.978	*P* = 0.001*

AUC: area under the curve; 95% CI: 95% confidence interval; HCC: hepatocellular carcinoma; DP.HCC: diagnostic prediction model for HCC. *Statistically significant (*P* < 0.05).

## Abbreviations

AUC: area under the curve
CI: confidence interval
DEA: differential expression analysis
DEGs: differentially expressed genes
DP.HCC: diagnostic prediction model for HCC
DP.MASLD: diagnostic prediction model for MASLD
FC: fold change
GEO: Gene Expression Omnibus
GO: Gene Ontology
GSEA: Gene Set Enrichment Analysis
HCC: hepatocellular carcinoma
H&E: Hematoxylin-eosin
ICGC: International Cancer Genome Consortium
KEGG: Kyoto Encyclopedia of Genes and Genomes
MASLD: metabolic dysfunction-associated steatotic liver disease
MASH: metabolic-associated steatohepatitis
ROC: receiver operating characteristic
RRA: robust rank aggregation
RT-qPCR: quantitative reverse transcription polymerase chain reaction
TCGA: The Cancer Genome Atlas
t-SNE: t-distributed Stochastic Neighbor Embedding
WGCNA: weighted gene correlation network analysis
