# Supplementary material for: Development of a Novel four-gene Model for Monitoring the Progression from Metabolic Dysfunction-associated Steatotic Liver Disease to Hepatocellular Carcinoma in Males
Source: J Cancer. 2025 Jan 1;16(3):917–31. doi: 10.7150/jca.100724 (PMC11705051; doi:10.7150/jca.100724)
Supplement: Supplementary file 1 — Supplementary figures and tables. [file jcav16p0917s1.zip › Supplementary Figure 1.pdf]

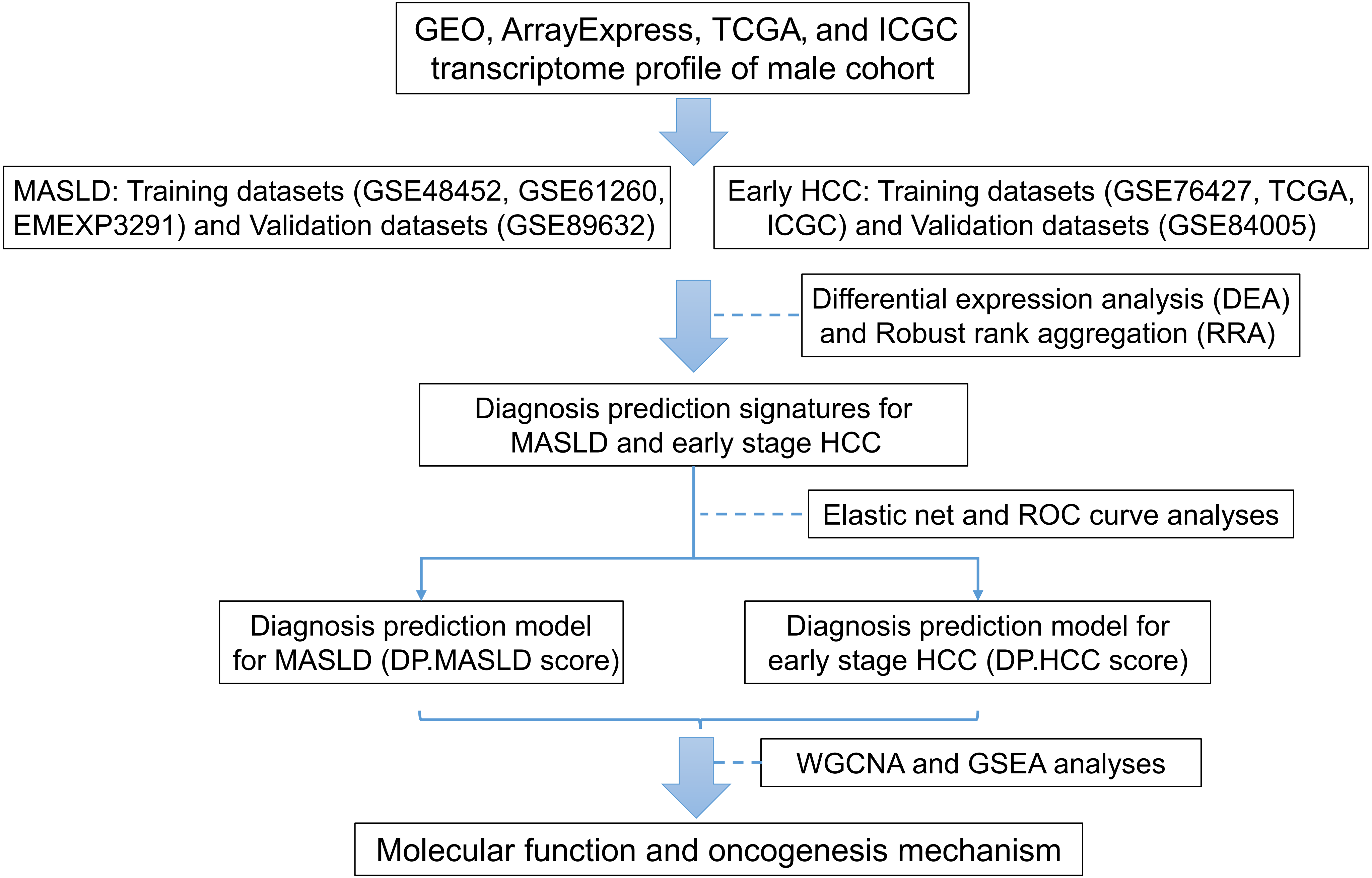
GEO, ArrayExpress, TCGA, and ICGC transcriptome profile of male cohort
MASLD: Training datasets (GSE48452, GSE61260, EMEXP3291) and Validation datasets (GSE89632)
Early HCC: Training datasets (GSE76427, TCGA, ICGC) and Validation datasets (GSE84005)
Differential expression analysis (DEA) and Robust rank aggregation (RRA)
Diagnosis prediction signatures for MASLD and early stage HCC
Elastic net and ROC curve analyses
Diagnosis prediction model for MASLD (DP.MASLD score)
Diagnosis prediction model for early stage HCC (DP.HCC score)
WGCNA and GSEA analyses
Molecular function and oncogenesis mechanism
