# Supplementary material for: Development of a Novel four-gene Model for Monitoring the Progression from Metabolic Dysfunction-associated Steatotic Liver Disease to Hepatocellular Carcinoma in Males
Source: J Cancer. 2025 Jan 1;16(3):917–31. doi: 10.7150/jca.100724 (PMC11705051; doi:10.7150/jca.100724)
Supplement: Supplementary file 1 — Supplementary figures and tables. [file jcav16p0917s1.zip › Supplementary Table 1.docx]

**Supplementary Table 1.** RT-qPCR primers for differentially expressed genes.

| Gene | Forward primer (5′ to 3′) | Reverse sequence (5′ to 3′) |
| --- | --- | --- |
| AKR1B10 | TCAGAATGAACATGAAGTGGGG | TGGGCCACAACTTGCTGAC |
| CYR61 | GGTCAAAGTTACCGGGCAGT | GGAGGCATCGAATCCCAGC |
| FABP4 | ACTGGGCCAGGAATTTGACG | CTCGTGGAAGTGACGCCTT |
| GNMT | CTGGGGTGGACTCCATTATGC | GATGACCCACTTGTCGAAGGC |
| TBHS1 | TCAGTGTCTCGACGAAGTAGT | TGGTTCCCATAGCCATCAGAG |

RT-qPCR: Quantitative reverse transcription polymerase chain reaction.
