# Supplementary figures and images for: Development of a Novel four-gene Model for Monitoring the Progression from Metabolic Dysfunction-associated Steatotic Liver Disease to Hepatocellular Carcinoma in Males
Source: J Cancer. 2025 Jan 1;16(3):917–31. doi: 10.7150/jca.100724 (PMC11705051; doi:10.7150/jca.100724)

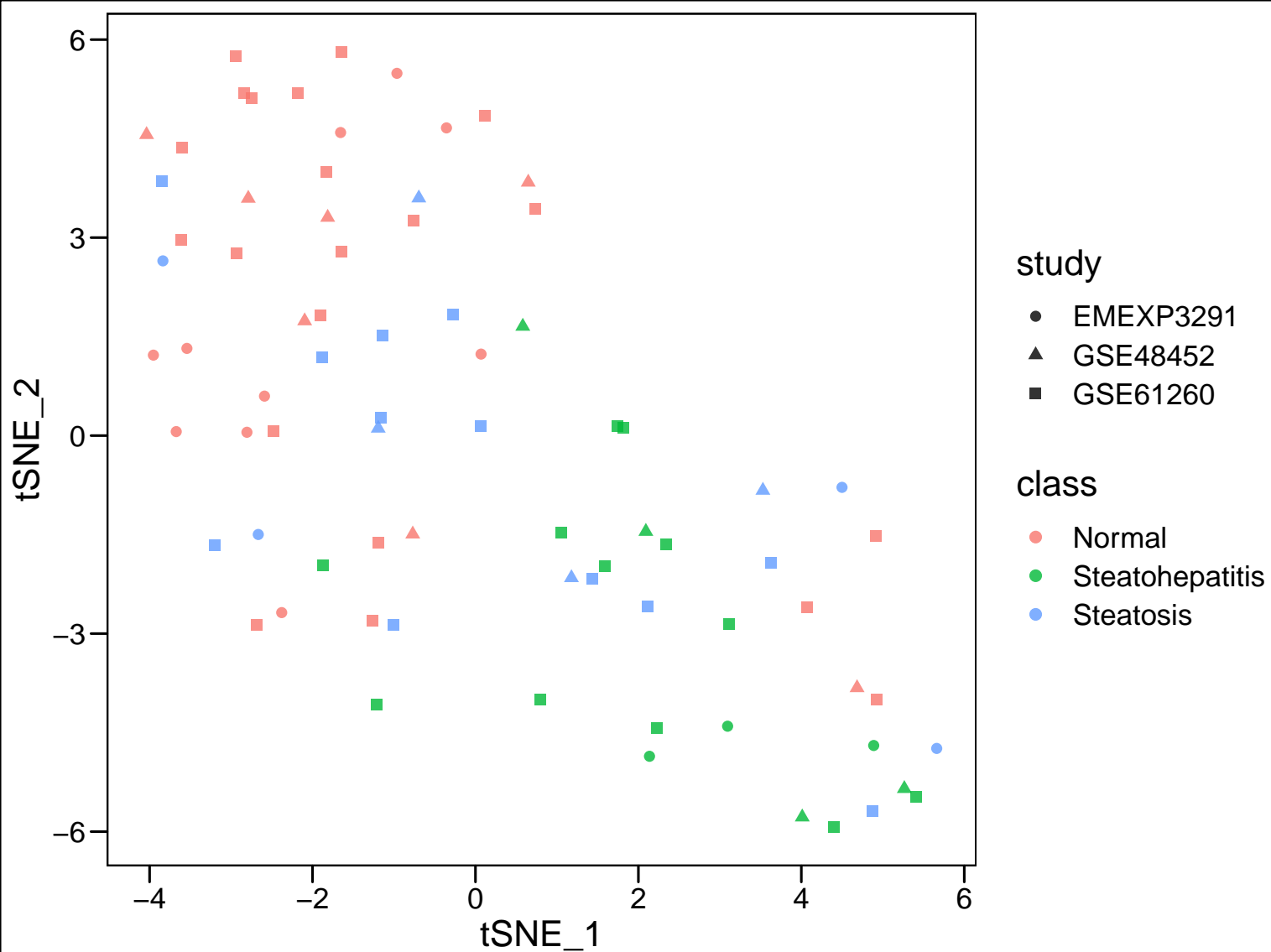

Supplement: Supplementary file 1 — Supplementary figures and tables. [file jcav16p0917s1.zip › Supplementary Figure 2.pdf]

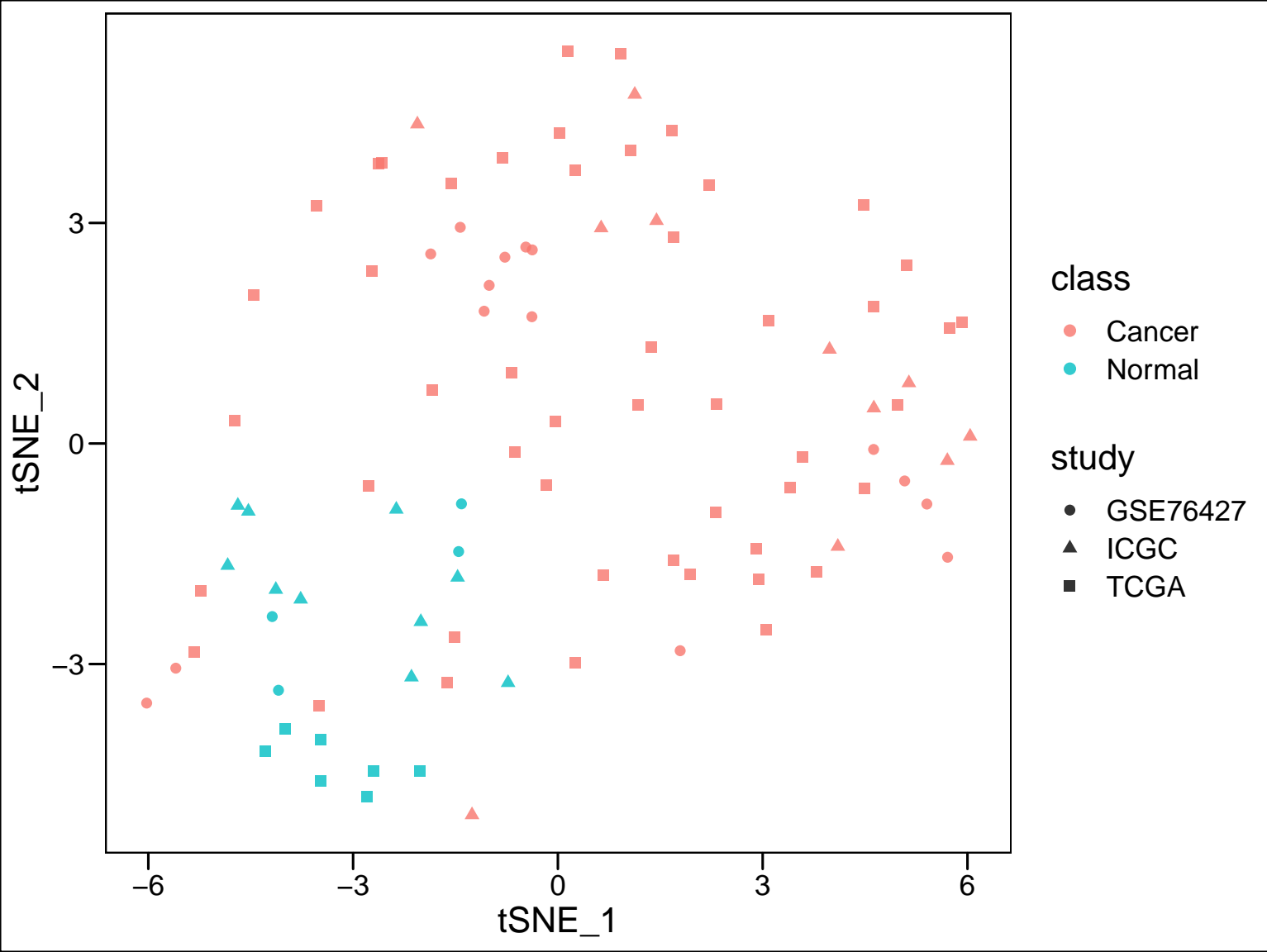

Supplement: Supplementary file 1 — Supplementary figures and tables. [file jcav16p0917s1.zip › Supplementary Figure 3.pdf]

# Cluster Dendrogram

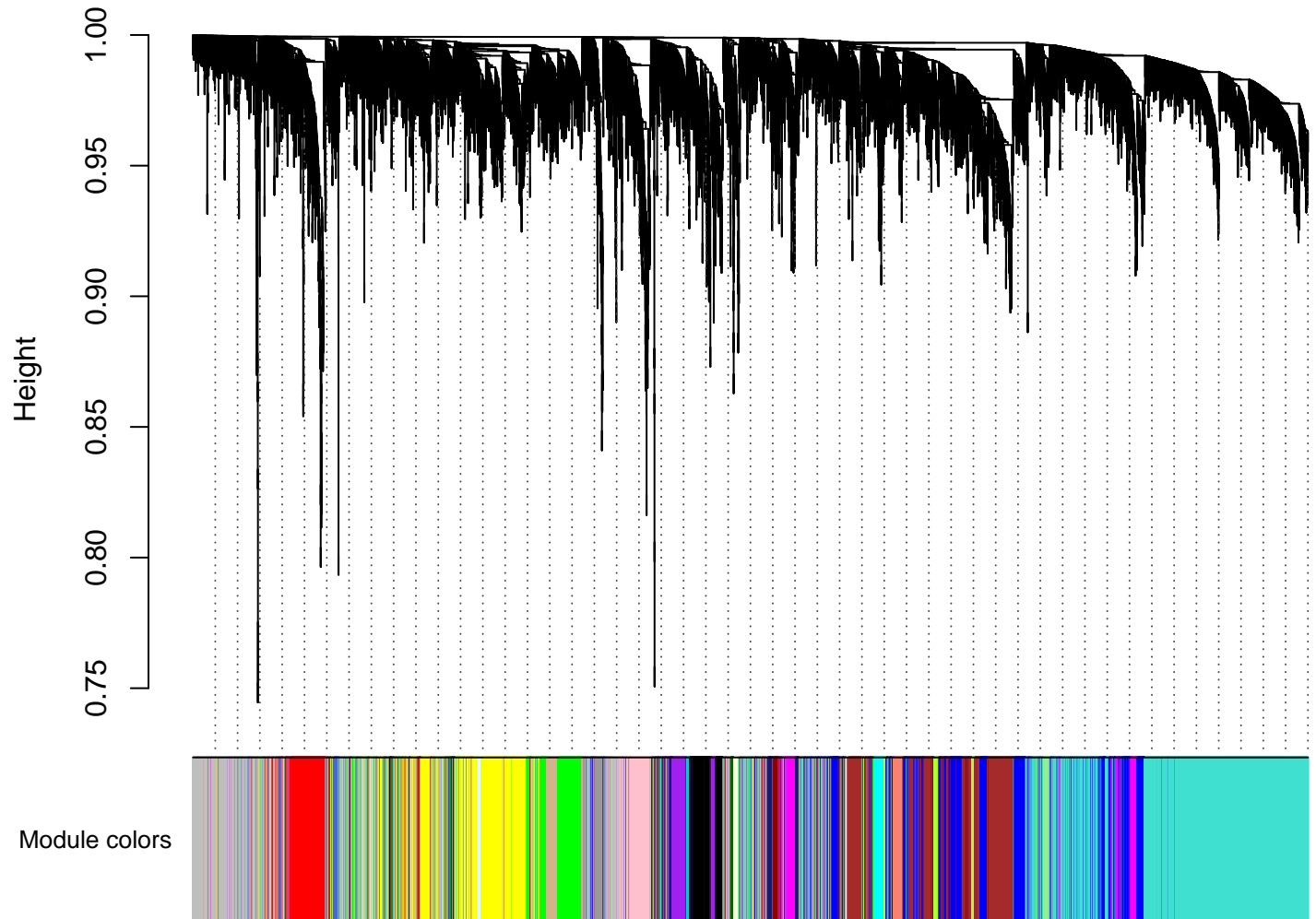

Supplement: Supplementary file 1 — Supplementary figures and tables. [file jcav16p0917s1.zip › Supplementary Figure 4.pdf]
